# Proteomic Approaches to Unravel Mechanisms of Antibiotic Resistance and Immune Evasion of Bacterial Pathogens

**DOI:** 10.3389/fmed.2022.850374

**Published:** 2022-05-02

**Authors:** Eva Torres-Sangiao, Alexander Dyason Giddey, Cristina Leal Rodriguez, Zhiheng Tang, Xiaoyun Liu, Nelson C. Soares

**Affiliations:** ^1^Clinical Microbiology Lab, University Hospital Marqués de Valdecilla, Santander, Spain; ^2^Instituto de Investigación Sanitaria Marqués de Valdecilla (IDIVAL), Santander, Spain; ^3^Sharjah Institute of Medical Research, University of Sharjah, Sharjah, United Arab Emirates; ^4^Department of Medicinal Chemistry, College of Pharmacy, University of Sharjah, Sharjah, United Arab Emirates; ^5^Division of Chemical and Systems Biology, Department of Integrative Biomedical Sciences, Faculty of Health Sciences, University of Cape Town, Cape Town, South Africa; ^6^Copenhagen Prospectives Studies on Asthma in Childhood, COPSAC, Copenhagen University Hospital, Herlev-Gentofte, Denmark; ^7^Department of Microbiology, School of Basic Medical Sciences, Peking University Health Science Center, Beijing, China

**Keywords:** system biology, host-pathogen interactions, PTMs (post-translational modifications), SWATH-MS, mass spectometry, antibiotic resistance

## Abstract

The profound effects of and distress caused by the global COVID-19 pandemic highlighted what has been known in the health sciences a long time ago: that bacteria, fungi, viruses, and parasites continue to present a major threat to human health. Infectious diseases remain the leading cause of death worldwide, with antibiotic resistance increasing exponentially due to a lack of new treatments. In addition to this, many pathogens share the common trait of having the ability to modulate, and escape from, the host immune response. The challenge in medical microbiology is to develop and apply new experimental approaches that allow for the identification of both the microbe and its drug susceptibility profile in a time-sensitive manner, as well as to elucidate their molecular mechanisms of survival and immunomodulation. Over the last three decades, proteomics has contributed to a better understanding of the underlying molecular mechanisms responsible for microbial drug resistance and pathogenicity. Proteomics has gained new momentum as a result of recent advances in mass spectrometry. Indeed, mass spectrometry-based biomedical research has been made possible thanks to technological advances in instrumentation capability and the continuous improvement of sample processing and workflows. For example, high-throughput applications such as SWATH or Trapped ion mobility enable the identification of thousands of proteins in a matter of minutes. This type of rapid, in-depth analysis, combined with other advanced, supportive applications such as data processing and artificial intelligence, presents a unique opportunity to translate knowledge-based findings into measurable impacts like new antimicrobial biomarkers and drug targets. In relation to the Research Topic “Proteomic Approaches to Unravel Mechanisms of Resistance and Immune Evasion of Bacterial Pathogens,” this review specifically seeks to highlight the synergies between the powerful fields of modern proteomics and microbiology, as well as bridging translational opportunities from biomedical research to clinical practice.

## Introduction

Mass spectrometry (MS) is an analytical approach used to measure the mass-to-charge ratio (*m/z*) of chemical compounds and calculate their exact molecular weight that was originally developed for determining the masses of individual atoms (1). Mass spectrometers consist mainly of three components: an ion source, a mass analyzer and a detector, usually an electron multiplier. The various combinations of these elements define, so far, a handful of different mass spectrometer types. First, the sample can be introduced directly into the mass spectrometer (direct infusion), or the sample analytes can be pre-processed and separated by gas or liquid chromatography (usually nano High-Pressure Liquid Chromatography) allowing for improved resolution and reduced sample complexity. The ion source is essential to ionize the neutral analyte into charged ions by gas phase methods, desorption, or spray. Broadly, gas phase methods such as electron ionization, chemical ionization, direct analysis in real time or inductively coupled plasma are not used in the microbiology field. While desorption methods, primarily laser desorption ionization (LDI), whether assisted by matrix (MALDI) or surface enhanced (SELDI) are used for clinical microbiology diagnosis, spray methods such as Electrospray ionization (ESI) or Desorption electrospray ionization (DESI) are better suited for microbiology research (1). Both desorption and spray methods are soft ionization techniques that allow for the analysis of large or labile molecules, yet MALDI suffers from the disadvantage of poor reproducibility due to the heterogeneity of the matrix-analyte crystals, and ESI/DESI cannot as yet be directly used for imaging (2). The five basic types of mass analyzer currently used in proteomic research are: (1) quadrupole (Q), (2) ion trap (IT), (3) orbitrap, (4) Fourier transform ion cyclotron resonance (FT-ICR), and (5) time of flight (TOF) instruments (Table 1). Along with their ability to determine the *m/z* of peptides, the main difference among the different types is their design and performance characteristics and whether ionized compounds are fragmented into smaller pieces. Mass analyzers can be stand alone or, in some cases, put together in tandem or in conjunction with ion mobility mass spectrometry, in which a counter flowing gas allows for the separation of ions with identical *m/z* but differing cross-sectional area.

**TABLE 1:** Description of main mass analyzers used in the field.

Mass analyzer	Advantages	Disadvantages	Uses
Quadrupole (Q) (3, 4)	• Very popular, easy to use and small and compact design • Durable and low cost • Reliable and long stability—good reproducibility and very good sensibility • Can be set to scan a specific *m/z* range or preselected masses to pass through the detector	• Need of a continuous flux of ions, which makes them less suitable for pulsed ion sources (e.g., MALDI) • Need a clean matrix to avoid the interference of unwanted ions • Limited mass ranges and resolution (not suitable for macromolecular compounds) • Low resolving power when used as single system and not in tandem	• Determination of standard mixtures and metabolites in cell and plasma extracts • Triple Quadrupole (TQ)-MS: three quadrupoles (Quad 1, Quad 2, and Quad 3) lined up in a row, is excellent for quantitative analysis commonly employed for routine targeted analyses (e.g., MRM, SRM)
Ion trap (IT) (3, 5)	• Small and compact design, as well as low cost • Good mass resolution • Better sensitivity than quadrupole analyzers • The ability to perform sequential fragmentation and thus derive more structural information from a single analyte (i.e., fragmenting an ion, selecting a particular fragment, and repeating the process) is called **MSn**	• Limited resolving power and narrow dynamic range • Upper limit on the ratio between a precursor’s mass-to-charge ratio (m/z) and the lowest trapped fragment ion (one third rule),—a significant limitation for the *de novo* sequencing of peptides • Fragmentation experiments in time rather than in space;—non-suitable for precursor ion comparisons	• Qualitative research of molecular structure, screening and protein identification.
Fourier transform ion cyclotron resonance (FT-ICR) (3)	• Very good accuracy—specially for low mass compounds • High resolving power and mass accuracy—the highest recorded mass resolution of all mass analyzers • Very high resolution and very good sensibility with a Stable mass calibration • Non-destructive ion detection—it does not require chemical or enzymatic cleavage for post-translational modifications (PTM) analysis	• Slow scan speed • Subject to space charge effects and ion molecule reactions • Requires a strong magnetic field and an extremely high vacuum; -requires maintenance of superconducting magnets • Artifacts may be found in the mass spectra • Expensive	• Empirical formulas can be obtained directly from mass data • Analysis of complex mixtures and molecular structures of large biomolecules • Well suited for use with pulsed ionization methods such as MALDI
Time-Of-Flight (TOF) (3, 6, 7)	• Fastest scanning and good sensitivity • Extremely high mass range (from few Daltons to well > 100 kDa) • Parallel acquisition of various *m/z* values	• Low resolution • Wide dynamic range and greater sensitivity, compared to a scanning instrument as a quadrupole. • Requires pulsed ionization method or ion beam switching, then usually it is coupled to modern ionization techniques like ESI	• Fast analysis of biological macromolecules and measure of the mass of many peptides simultaneously • Analyzer of choice for analyzing high mas biomolecules like proteins, and peptide mass fingerprint analysis • Good for kinetic studies of fast reactions and for use with gas chromatography to analyze peaks from chromatograph • In a reflectron mode the resolution is increased without dramatically losing sensitivity or needing to increase the size of the flight (or drift) tube • The TOF/TOF system provides faster and most confident identification and relative quantitation of proteins;—ideal platform for biomarker discovery, MALDI MS imaging, and protein identification.
**HYBRID MASS SPECTROMETERS**			
QToF (3, 8–10)	• This pairing combines accurate mass measurement, the ability to carry out fragmentation experiments, and high-quality quantitation. • In the QTOF, precursor ions are selected in the Quadrupole and sent to the collision cell for fragmentation. The generated product ions are detected by TOF • High mass resolution and wide mass range • High-resolution spectra and high sensitivity • Medium dynamic range of detection	• High cost • Requires careful maintenance.	• Qualitative analysis with a precise molecular weight and identification of degradation products • Structural elucidation • Sequencing; -identification and analysis of amino acid sequences • Q-TOF can be used with MALDI and ESI, both suitable for biomolecules such as proteins
Q-Trap (QT) (3)	• The increased volume of a linear trap instrument (over a three-dimensional ion trap) improves dynamic range • Wide dynamic range of detection • Highest sensitivity • Lower cost	• Low mass resolution	• Suitable for SRM or MRM • Quantitative and targeted analysis • Suitable for complex biological samples, and especially useful to measure small molecule disease markers in complex clinical samples
IT-TOF (3)	• The 3D IT is used as a mass selector and reactor, combining the multistage MS capability of the IT and the high-resolution capability of the TOF • Multistage MS (MS*^n^*) • High mass resolution	• Limited in scan modes • Sensitivity to mixed samples is not good • Weak quantitative ability.	• Qualitative analysis • Structural elucidation • Sequencing • Characterization of glycoprotein
Orbitrap (3, 11, 12)	• Hybrid ion trap/FTMS (FT-ICR or Orbitrap) • Small and little maintenance compared to FT-ICR (no need of superconducting magnets) • Precursor ions are selected and fragmented in an external ion trap. The generated product ions can be detected either in the external trap (lower mass resolution, but faster) by or by FTMS (higher mass accuracy and resolution, but slower) • Extreme capability of measuring mass with the ability to resolve closely related masses • High resolving power	• High cost	• Good for low mass compounds • Screening of complex samples and compound confirmation • The hybrid mass spectrometers, such as the Q-Orbitrap and Q-Orbitrap-IT use high-capacity multipole ion traps to accumulate ions before analysis. The Q-Orbitrap-IT mass spectrometer is used to perform accumulated ion monitoring for targeted proteomics

In the last decades, MS has increasingly become the method of choice for analysis of complex protein samples, making MS-based proteomics the gold standard for large-scale determination of gene and cellular function directly at the protein level (13). Proteomics revolves around the identification and quantification of the full protein complement (known as the proteome) of a cell, tissue, or an organism (typically *via* peptides). The proteome is highly dynamic due to complex regulatory systems that control the expression levels (14), location (15), activity and conformation of proteins, resulting in significant differences of cell/organism response to any given stimulus. As a result, proteomic technologies have been useful and widely practiced in modern biomedical research, including studies of various bacterial pathogens.

Traditionally, microbiologists rely mostly on genetic approaches (e.g., assess the phenotype(s) upon knocking out a gene). Indeed, such methods have contributed to most of our understanding of basic biology associated with bacterial pathogens. For instance, it is a common scenario that not all mutants would exhibit an observable phenotype, thus leaving no clues for deducing the function of the gene of interest. Therefore, proteomics offers an important alternative that complements the traditional reductionist approach. With the measurements of thousands of proteins all at once, proteomic studies afford a comprehensive and, more importantly, quantitative view of protein constituents of bacterial pathogens. When such data are collected under different physiological conditions, one can analyze the quantitative information associated with all expressed proteins and understand those altered pathways/processes that are engaged in antibiotic resistance, virulence, etc. In addition to expression studies, protein modifications, localization and protein-protein interactions can also be examined in such a high-throughput fashion. Here, in this review, we summarize the various applications of proteomic tools for understanding the biological mechanisms of bacterial pathogens as well as bacteria-host interactions.

## Modern Proteomics in the Microbiology Sequencing ERA

**FIGURE 1:**
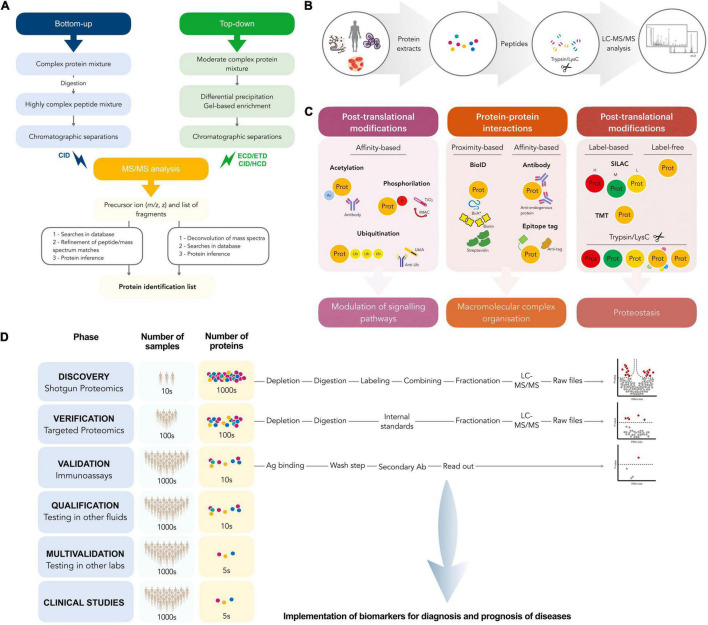
**(A)** Work-flow followed in bottom-up and top-down proteomics. **(B)** A schematic workflow for prototypical bottom-up proteomics experiments. Protein lysates from infected biological samples, are digested by trypsin and/or LysC, and the resulting proteolytic peptides are analyzed by LC-MS/MS. **(C)** Applications and main features of different bottom-up proteomics workflows, label-free or label-based SILAC, and tandem mass tag MS methods applied for global proteome profiling. Workflows can be also coupled with affinity purification-based methods to elucidate host-pathogen PPI networks. **(D)** Workflow for discovery and validation of biomarkers.

Since the introduction of MS-based methods in proteomics, especially that of tandem MS/MS (MS2), the technology has rapidly accelerated to a state in which large scale analysis with high mass accuracy and resolution, wide proteome coverage and accurate quantitation is routinely achievable. MS-based proteomics typically follows one of two strategies: bottom-up or top-down. Top-down proteomics deals with the analysis of intact proteins while bottom-up deals with the analysis of peptides resulting from protein enzymatic digestion and the subsequent inference of the originating proteins (Figure 1A). Historically, protein and peptide sequencing were accomplished by means of Edman degradation since its discovery by Pehr Edman in 1950 (16–18). With predictable peptide fragmentation by collision induced dissociation (CID) and higher energy collision induced dissociation (HCD) among others, as well as improved knowledge of fragmentation rules, it has been possible to reconstruct peptide sequences given high quality MS2 spectra and the precursor masses and this is the domain of *de novo* peptide sequencing (19–22). However, *de novo* sequencing algorithms can be demanding of computational performance and exacting with respect to MS2 spectra quality and MS resolution—originally orders of magnitude less than what is commonly available today (23, 24). In addition, *de novo* peptide sequencing for large-scale proteomics remains challenging because of the lack of full coverage of ion series in tandem mass spectra (25). The subsequent development of peptide sequence tags (26) and database search algorithms like SEQUEST (27) or MASCOT (28) and those that followed [Crux (29), Comet (30), and Andromeda (31)] have made peptide identification possible even on common laptop computers and accessible to researchers of varying levels of familiarity with the underlying mathematics. Although some existing new methods improved the performance of *de novo* sequencing (algorithms, such as PEAKS (32), PepNovo (23, 33), or machine learning/big data approaches) the precision and throughput were still far lower than expected.

Bottom-up, or shotgun proteomics, provides simpler, more generalizable chromatographic separation than can be achieved with top-down approaches, as short peptides are less diverse in chemical attributes (e.g., hydrophobicity or pI) than larger proteins. Ultra-high pressure reversed phase liquid chromatography (uHPLC, often with C18 beads as the stationary phase) utilizing a water—acetonitrile gradient as the mobile phase system is a common choice for the chromatographic system. Most biofluids, from urine (34) to blood (35) to CSF (36) to bacterial biofilms (37), cell samples (38) or even soil (39) and waste-water samples (40) can be processed along similar principles: The sample is homogenized and the protein extracted, digested, desalted, and analyzed by the appropriate MS system, typically with inline (i.e., with the fractionated sample being ionized for analysis continuously as eluted) chromatographic separation and often with some enrichment step or pre-fractionation where applicable or needed (Figure 1B). However, there remain many deviations from this general rule with direct sampling by MALDI-MS2 for added tissue-level spatial information being one that differs in nearly every aspect listed before, and one being used ever increasingly.

Conversely, in top-down methods intact proteins are analyzed, making these workflows particularly suited for the analysis of distinct and co-existing post-translational modifications (PTMs) of a protein or protein complex (41, 42). Some of the major advantages to top-down approaches include largely eliminating the problem of protein inference and preserving higher-level organizational information (43). However, this approach typically requires some form of charge state stabilization or extremely high *m/z* resolution from the instrument (44). Often, top-down approaches are combined with tandem MS methods to distinguish between protein species and various modifications can be both located and identified (41).

MS-based proteomics even allows for the determination of 3D protein structure. By implementing ion mobility separation in the MS analysis, additional information about the 3D structure of proteins and protein complexes, such as collision cross-sectional area, can be obtained (42, 45). Crosslinking mass spectrometry (XL-MS) is a specific covalent labeling (CL)-MS approach to study protein complexes, in which crosslinking agents link nearby sidechains (solvent-accessible amino acids); the labeled residues can be compared in different conditions, inducing a conformational change. Based on the length of the crosslinker it is possible to create 3D models from the resulting distance maps (46). The technique provides sufficient structural data to compute 3D models (47) and can elucidate structural models of large protein complexes reaching atomic resolution, as well as peptide level (48, 49). Regarding the topic of this review, *in vivo* XL-MS is a promising tool to discover virus–host associations and pathways (50), as well as potential interfaces for drug design or to derive a drug’s mode of action. With regards to drug discovery, nuclear magnetic resonance (NMR) and X-ray crystallography are still the structural methods of choice in a hit-to-lead or lead optimization strategy, however, native MS and hydrogen–deuterium exchange have been used as alternative methods and are regularly employed by biopharma companies (41).

There are generally two quantitation strategies to consider with MS-based proteomics experiments: label-dependent or label-free (Figure 1C). Given that compound isotopes are nearly indistinguishable chemically but yet differ in mass, MS experiments allow to distinguish them with minimal risk of effecting any biological interpretation. This enables the intentional “labeling” of samples by stable, non-radioactive isotopes to allow for subsequent mixing of the samples and thus allowing distinct samples to undergo all subsequent handling steps, with chromatographic separation and ionization together for more accurate quantitative comparison between samples. This labeling can be performed enzymatically during sample processing using heavy-labeled (18O) water during digestion (51), or chemically, using heavy isotoped dimethyl-labeling of free amine groups (52), or metabolically. An example of the latter is Stable Isotope Labeling of Amino acids in Cell Culture (SILAC), where cells are grown in media substituted with either exclusively light or heavy isotopes of a subset of amino acids, causing all peptides containing those amino acids to be detected at either the usual (lighter) *m/z* or shifted by the precise mass difference between the heavy and light amino acid isotopes provided to the growing cells (53, 54). The earlier that samples can be combined, the more that the technical variation or error can be generalized among samples, which consequently improves the power to detect differences between the samples. As such, metabolic labeling methods such as SILAC provide the best comparative quantitation between samples. Nevertheless, the downsides of this technique include high costs of isotopically pure cell culture substitutes, and low numbers of distinct isotope labels with “light,” “medium,” and “heavy” being the present limits. Additionally, combining “light” and “heavy” samples in one analysis results in doubled sample complexity at the precursor or MS1 level and that can result in fewer peptide species being identified, although this can be mitigated by pre-fractionating the combined samples. The related technology of isobaric tag for relative and absolute quantification (iTRAQ) allows for combining up to 16 samples in a single analysis and does away with the increased MS1 complexity by balancing the isotope weights on either side a labile chemical bond such that the overall tag masses are identical at the MS1 level, but distinct at the MS2 level upon fragmentation. However, this labeling technique is applied later than SILAC in the traditional sample preparation workflow and cannot account for experimental variation prior to labeling.

In contrast to isobaric labeling there is also label-free quantitation (55, 56). In this strategy samples are combined only at the post-acquisition data level, and all biological and technical variation are included in the final measure. This strategy is dependent on reproducible sample handling, chromatography and ionization conditions and benefits from large sample numbers. Given the limiting numbers of samples able to be multiplexed *via* labeling methods and the financial cost of doing so, label-free quantitation is preferable for large clinical studies or settings where costs may be limiting. Post-acquisition data normalization is typically performed and software and algorithms specific to label-free proteomics have been developed (57, 58).

MS-based proteomics has replaced the classical 2D gel electrophoresis, allowing instead for a highly quantitative detection technology in liquid chromatography coupled to electrospray triple-quadrupole mass spectrometer. However, MS-based proteomics analyses can follow either the broadly discovery shotgun approach in which thousands of peptides are detected and quantified or the highly sensitive and reproducibly quantitative targeted approach. Selected reaction monitoring (SRM), also denominated multiple reaction monitoring (MRM), is often used to analyze a complex mixture of tryptic peptides to quantify a specific and preselected subset of the given mixture (59, 60) and its primary advantage is the ability to detect multiple isoforms and PTMs with high specificity, reproducibility and sensitivity, and accurate quantitation in a single test run (61). Parallel reaction monitoring (PRM) is an SRM-like targeted acquisition method based on the quadrupole-orbitrap (Q-orbitrap) mass spectrometer, being both (SRM and PRM) widely used for the discovery and validation of biomarkers (Figure 1D).

On the other hand, data-dependent acquisition (DDA)-MS is widely used to generate high-quality references of peptide fingerprints and is the basis of most discovery, or shotgun, proteomics experiments, allowing for the quantitation of thousands of peptides in a single analysis. However, this approach suffers from the stochastic selection of abundant ions that impairs sensitivity and reproducibility. Otherwise, in data-independent acquisition (DIA)-MS, the systematic fragmentation and acquisition of all fragment ions within given isolation *m*/z windows yields a comprehensive map for a given sample. However, most DIA approaches require comprehensive DDA-based spectral libraries, the development of which can sometimes become impractical for studying non-canonical and personalized neoantigens (62). New DIA approaches, such as sequential window acquisition of all theoretical mass spectra (SWATH-MS) combine the benefits of both targeted and shotgun approaches to provide high-throughput, accurate quantification and reproducible measurements within a single experimental setup (59, 63–65). SWATH-MS has become a useful methodology in the context of drug or vaccines development (66), identification of biomarkers of HIV-1 (67), evaluation of changes in tissue-specific protein profiles in sepsis (68), evaluation of changes in the proteome under antibiotic pressure (69) and providing novel insights from central nervous system (CNS) functioning and the host response with meningitis (70).

## Microorganisms and their Proteomes

Genetic variability is an evolutionary bacterial mechanism of adaptation to avoid and escape from both the immune system and antibiotic pressure (i.e., by developing resistance to antibiotics). Quantitative proteomics can provide accurate molecular phenotypes of microbes, which are difficult to determine using alternative technologies (71). Thousands of proteins interact through physical association and PTMs to give rise to the emergent functions of cells. The comprehension of these functions requires the proteomes’ study as co-ordinated “systems” rather than merely collections of individual protein molecules. The interacting proteome or “protein networks” provides the ability to see the proteome as a whole, mostly represented by network models: proteome-wide physical protein–protein-binding interactions organized into Protein Interaction Networks (PINs), and proteome-wide PTM relations organized into Protein Signaling Networks (PSNs) (72). Thus, proteomics serves us as an excellent tool for comparing microbial proteomes from the same family with different virulence or phenotypic factors, as well as for studying the interactions between pathogen and host proteins that allow the hijacking of the host’s transcriptional and translational machinery (73).

The properties and behavior of the proteome as an integrated system sheds light on complex biological processes and phenotypes encompassing numerous biological processes which are concurrently and co-ordinately active in every living cell (74). A big challenge for Clinical Microbiology is to understand the mechanisms determining whether a bacterium is a threat or is simply part of our microbiota. For example, *Staphylococcus aureus* is well-known as a human pathogen, as well as part of the skin and mucosa flora. Most of the *S. aureus* adaptive reactions to “new” environments require complex changes in transcription, protein expression and metabolism (75). The importance of chromosomal background is stressed by genome-scale metabolic reconstructions showing that bacteria can be categorized as pathogenic or commensal according to their metabolic capabilities. For example, the relative protein levels between enterotoxigenic *Escherichia co*li (ETEC) and BL21 *E. coli*, indicated that the ETEC strains share certain metabolic functions that can be favorable for successful gut colonization, but additionally produced considerably higher levels of proteins that can generally enhance bacterial pathogenicity. These characteristics appeared to be shared by most of the ETEC strains, but individual differences demonstrated heterogenicity in the amounts of specific metabolic proteins (76). However, the why and how of these shifts have begun to be unraveled and even measured by the hand of “omics” technologies, especially proteomic approaches (77, 78). As a note of this adaptation, when the bacterium meets its host, a cascade of intracellular interactions shapes the outcome of the infection through alterations in protein abundance, localization, and PTMs (79).

Currently, the ability to readily study the proteome of bacteria grown on solid media has made possible new procedures such as single colony cell proteome analysis and identification of various adaptation and resistance mechanisms. The proteomic analysis of bacterial isolates grown on solid media is gaining increased interest from researchers to explore the proteomes, “*in situ*,” with minimally passaged isolates such as those used in primary culture plates. Fortuin et al. reported 1,650 protein groups from *E. coli* K12, identifying unique proteins involved in key metabolic processes missed in liquid culture that could be further investigated for its involvement in pathogenesis and virulence (80). Using a simple workflow, they also characterized proteins implicated in swarming motility, influencing the spreading of bacterial cells on a surface, providing a unique insight into the differential expression of key virulence proteins within biofilm-like microenvironments in single colonies (81).

As discussed above, the recent development of quantitative proteomic methods such as DIA-MS has allowed for accurate quantification of proteins with a high degree of data completeness and dynamic range without previously specified target peptides (82). Happonen et al. used DIA-MS for the first time for identifying dynamic protein interactions from the interface between *Streptococcus pyogenes* and the human host and determined those interactions which are crucial for immune evasion and phagocytosis (83). Similar methods such as SWATH-MS have also proven valuable for the quantification of *Mycobacterium tuberculosis* over different conditions as well as for the analysis of PTMs (84). Protein XL-MS and computational modeling has also gained attention for unveiling protein interactions. Early this year Birk et al. developed another new method combining metabolic and chemical isobaric peptide labeling to simultaneously determine time-resolved protein decay and *de novo* synthesis in *Listeria monocytogenes* (85). Employing this new method, the authors identified more than 100 ClpC target proteins and observed indirect effects of the *clp*C deletion on the protein abundance in diverse cellular and metabolic pathways, highlighting the crucial role of ClpC for *L. monocytogenes* adaptation to the host environment through proteome remodeling.

Examples of the use of proteomic methodologies in disentangling host-pathogen interactions include studies focussed on the proteomics of the pathogen cell-wall as the major interface (86, 87), analyses of intracellular host proteomes during infection (38) and analyses of secreted pathogen proteins, among others. Using combinations such as protein cross-linking with MS and computational modeling or affinity purification strategies with SWATH-MS, all integrated in the system biology frame are useful to unravel the networks formed during these host-pathogen interactions too. Recently, multiple proteomic studies identified 13 proteins reproducibly differentially expressed in patients with COVID-19, which were related to cytokine-cytokine interaction, IL18 signaling, fluid shear stress and rheumatoid arthritis, which together validated prior indications of the involvement of cytokine storms in COVID-19 cases (88).

## The Dynamic Host-Pathogen Interactions During Infection

During infection, both bacterial pathogens and host cells reprogram their gene expression, which shapes the delicately balance of host-pathogen interactions. These host–pathogen interactions are highly dynamic across all stages of pathogenic infection, and the immune system’s response to pathogen-associated molecular pattern (PAMP) activation of immediate host inflammatory and antimicrobial responses (89). Direct measurement of dynamic protein abundance (i.e., expression profiling) from both bacteria and host cells in infection models, therefore, offers unique holistic insights into those molecular mechanisms underlying bacterial pathogenesis (90, 91). In this regard, studies of proteomic profiling of bacteria and other intracellular pathogens recovered from infected host cells, seems to lead that of studies examining the mammalian host, partly due to the relatively compact size of bacterial proteomes. Proteomic analyses of intracellular bacteria are often prone to host contamination (92). Various approaches have been developed to physically isolate bacterial cells from their host contaminants, including FACS sorting of fluorescently labeled bacteria (93) and differential centrifugation upon mild lysis of host cells (94). With those methods cautiously practiced, one can harvest intracellular bacteria with minimal host proteins, thereby rendering them amenable to proteome readout with sufficiently high coverage (95, 96).

As a gram-positive bacteria *Streptococcus pyogenes* has been proposed as a model by which to understand the different immune responses within a population against a common pathogen and its main virulence factors, such as the different subtypes of M protein (83, 97, 98). The diverse domain arrangement and high sequence variability of the M proteins enable *S. pyogenes* to form protein interactions with various human proteins, revealing a dense and highly organized protein interaction network (99). To determine the stoichiometric relationship between pathogen, surface proteins, and interacting host proteins, the Malmström group developed a dynamic model to study the relationship between the bacterial surface and its adhered host proteins (100) by a surface adsorption plasma approach in combination with MS (99, 101). The same Malmström group determined the Fc-binding interface, demonstrating a specific site in the IgG CH3 domain (essential for binding to FcγR receptor). Mimicking an *ex vivo* scenario during invasive infections, these interactions revealed binding with the non-immune Fc-domain, locking the FcγR receptor interaction, and assisting the bacteria in evasion from phagocytic killing (102).

Khakzad et al. developed an affinity procedure preceded by a chemical cross-linking on human blood plasma using live *S. pyogenes* to characterize the multicomponent human complement system membrane attack complex (MAC) associated with the bacterial surface and provided detailed information of protein–protein complexes in their native environment (103). In the same line, the same authors, early this year combined a targeted XL-MS approach with molecular dynamics (MD) simulations to characterize the *S. pyogenes* M1 protein (virulence factor) and human-IgG interactions (102). Accordingly, XL-MS revealed binding interfaces, while the MD simulations helped to elucidate the interaction network in molecular detail. The authors revealed important peptides, at the binding interface of the bacterial M1 protein in complex with human IgGs, playing a crucial role in the interactions. Certainly, XL-MS is becoming a routine tool for protein structure determination, conformation analysis, and mapping protein interactions in complex mixtures, as well as intact living cells (104, 105), unveiling the structural mechanisms of immune response and bacterial evasion by using a combination of mass spectrometry acquisition techniques such as DDA, DIA, and high-resolution MS1 (hrMS1) (102, 103).

As a Gram-negative bacterium, *Salmonella enterica* serovar Typhimurium (referred to as *Salmonella* hereafter) is arguably the most studied model pathogen at the proteome level, and as such we will discuss several proteomics examples related to this pathogen. Back in 2006, intracellular *Salmonella* from infected RAW 264.7 macrophages were analyzed, though only a fraction of its proteome (∼300 proteins) was measured (106). Later, the Liu group managed to expand the proteome coverage to nearly 2,000 bacterial proteins by optimizing conditions for cell lysis and differential centrifugation. The resulting large-scale proteomic datasets revealed extensive adaptations (i.e., metabolic remodeling as well as differential regulation of virulence factors) of the intracellular pathogen to host epithelial cells (95). Upon breaching the intestinal epithelial barrier, *Salmonella* can further spread to other organs *via* macrophage cells. Recently, the Liu group made the first attempt to directly contrast intracellular *Salmonella* proteomes within distinct types of host cells (i.e., macrophage vs. epithelial cells) (107). Though many features were shared, proteomic remodeling exhibited substantially faster kinetics in macrophages than in HeLa cells. Intriguingly, exceedingly high levels of histidine biosynthetic enzymes were observed exclusively in RAW 264.7 cells but not in epithelial cells. Yet subsequent experiments found much lower levels of histidine in RAW 264.7 cells than was in HeLa cells. Strikingly, it was found that bacterial hypersensitivity to host histidine deficiency is mostly attributed to the mutation of *hisG*, which encodes an essential enzyme in histidine biosynthesis.

In addition, intracellular bacterial proteomics has been applied to the characterization of mutants. For example, the Hensel group analyzed the intracellular proteome of two *Salmonella* mutants, Δ*ssaV*, and ΔsseF, in order to investigate the functional roles of *Salmonella*-induced filaments (108). And the Liu group also profiled the intracellular proteome of a *Salmonella* mutant lacking a putative regulatory gene, *ydcR*, which was found to transcriptionally control the expression of a known virulence factor, SrfN (109). Other than *Salmonella* typhimurium, a number of groups also examined the proteome of other bacteria residing within host cells, such as *Shigella flexneri* (110), *Listeria monocytogenes* (85), *Bordetella pertussis* (111), *Brucella abortus* (112), and *Staphylococcus aureus* (113). Last but not least, there have also been attempts to study bacterial proteomes from *in vivo* infections (93), though thus far such practice can still be technically challenging mostly due to limited amounts of sample recovered from infected animal tissues.

In comparison, there are relatively fewer studies of host cells with respect to expression profiling in infection models. Typically, extensive (and tedious as well) sample fractionation would be required to achieve comprehensive coverage of the host proteome in LC-MS measurements. Nevertheless, studies on changes to the host proteome during infection have been done. In terms of *Salmonella* infection, for instance, the host proteomic responses have been studied in both macrophage (114) and epithelial cells (115). Recently, the Typas group quantitatively measured newly synthesized host proteins during *Salmonella* infection as well as in specific cellular organelles (116). Importantly, their proteomic findings led to the discovery that cathepsin plays a role in non-canonical inflammasome regulation. As a matter of fact, currently we may gain more insights into infection biology by examining sub-proteomes with less complex samples until further major advancements in proteomic sequencing speed.

## Post-Translational Modifications and Other Protein Modifications During Infection and Antibiotic Therapy

PTMs regulate numerous biological processes in host-pathogen interactions. Several common PTMs, such as phosphorylation and ubiquitination, can now be examined on a proteome-wide scale. For example, global phosphoproteomic profiling can be routinely carried out by combining highly efficient phosphopeptide enrichment strategies with quantitative LC-MS measurements. In 2011, the Foster group carried out the initial phosphoproteomic study of *Salmonella*-infected epithelial cells and reported close to 2,000 phosphorylated host proteins (117). Furthermore, they expanded such analyses to host cells infected by a *Salmonella* mutant (Δ*ssaV*) to assess the overall influence of SPI-2 effectors on host phosphorylation pathways (118). Notably, they found HSP27 as a kinase substrate of the type III effector SteC. Very recently, the Dikic group also performed global profiling of host phosphoproteome upon *Salmonella* infection (119). Interestingly, they identified the host kinase SIK2 as a central player in the host defense against bacterial infection by mediating the actin cytoskeleton architecture around the *Salmonella*-containing vacuole (SCV). A few years ago, this group also measured the global ubiquitination events of *Salmonella*-infected host cells (120). In addition to several pathways with altered ubiquitination levels, they found infection-induced ubiquitination mediates the activity of CDC42 as well as linear ubiquitin chain formation, both of which are required for NF-κB activation.

It is well established that many bacterial pathogens express and secrete dedicated virulence factors, which have the capacity to catalyze PTMs of their host targets. However, due to weak enzyme-substrate interactions in general, it can be rather challenging to discover the host targets of these bacterial enzymes in the first place. Instead, the landscape of host PTMs (with and without the expression or delivery of bacterial enzymes) can be quantitatively profiled, as a powerful screening method for identifying the enzymatic substrates of bacterial effectors. For example, *Salmonella* type III effectors SseK1 and SseK3 possess glycosyltransferase activity that can catalyze the covalent attachment of N-acetyl glucosamine (GlcNAc) to protein substrates. By utilizing an antibody that specifically recognizes GlcNAcylation, two independent studies applied host glycoproteomics to screen for the enzymatic substrates of SseK1 and SseK3 (121, 122). From these studies, a few host targets were successfully identified which included the signaling proteins TRADD, TNFR1, TRAILR, and several Rab GTPases.

Other than the proteome wide PTM profiling as discussed above (i.e., for those modifications with enrichment tools), considerable efforts have also been made to discover non-canonical PTMs catalyzed by bacterial virulence factors. A paradigm in this field is the exquisite modifications of host small GTPase Rab1 by multiple *Legionella pneumophila* type IV effectors (123). A number of groups, have contributed to the discovery of at least four covalent modifications of Rab1 including AMPylation (124–126), phosphocholination (127, 128), non-canonical ubiquitination (129), and glucosylation (130). Some of these modifications are completely reversible and the de-modifying reactions are also catalyzed by dedicated bacterial effectors. In the studies above, mass spectrometry has played an indispensable role in the characterization of these protein modifications, for those previously undocumented PTMs. Very recently, in collaboration with the Shao group, the authors uncovered an unprecedented protein modification (dubbed ADP-riboxanation) mediated by *Shigella flexneri* type III effector OspC3 (123). Remarkably, this novel biochemical reaction couples the canonical ADP-ribosylation with an extra step of deamination. Functionally, OspC3-catalyzed modification of caspase-11/4, a central component of the pyroptosis pathway, inhibits bacterial lipopolysaccharide (LPS)-induced activation of host inflammatory death. Mechanistically, ADP-riboxanation on Arg314/310 of caspase-4/11 blocks their autoprocessing as well as the subsequent recognition and cleavage of GSDMD, the pore-forming protein that executes pyroptosis.

As a final note, though not as prevalent as their mammalian counterparts, PTMs of bacterial proteins are also worthwhile studying in the context of host-pathogen interactions. In fact, the methodology and its practice have been reported for global analyses of protein acetylation in *Salmonella* (131, 132). The Blackburn lab were able to observe novel mechanisms of vitamin C induced dormancy in the phosphoproteome of mycobacteria (133), and the Maček lab routinely analyze the phosphoproteome of bacteria (134).

## Mass Spectrometry Characterization Applied to Infectious Diseases

Early diagnosis can mean the difference between life or death for a patient. As a clear example, sepsis is a serious medical condition which can cause organ failure and death, with only a 30–40% positive detection rate from blood culture (135). Yet the early diagnosis or detection/identification of sepsis is key to preventing its progression to severe sepsis and septic shock. However, the lack of specific biomarkers that can differentiate sepsis from non-infectious systemic inflammatory diseases often leads to excessive antibiotic treatment (135, 136). Mass spectrometry and “omics” strategies allow for untargeted profiling of thousands of proteins and metabolites from human biological samples obtained from septic patients. Differential expression of, or modifications to, these proteins and metabolites provides a more reliable source of diagnostic biomarkers for sepsis (135, 137).

The identification of a “good” disease-specific biomarker enables the more accurate early diagnosis and prognosis of disease (138). In the post-genomic age, MS based proteomics reaches toward complete proteome coverage in humans and other organisms, both producing and drawing upon large quantities of information and bioinformatics resources (61). Proteomics-based approaches enable the elucidation of those biomarkers actually expressed from the potential encoded in the genome sequence information, to identify disease-specific protein and peptide biomarkers which could not be predicted from the genome alone (138, 139). However, the bacteria follow certain growth laws which balance energy flux and protein synthesis and show great flexibility in regulating the expression of proteins needed for a particular environment. Thus, the growth conditions ultimately influence the levels of produced proteins, resulting from specific nutrient availability together with differential expression (140), and so make meaningless the idea of biomarkers for bacteria independent of their growth environment.

Blood plasma, serum, sputum/saliva, and urine are common and relatively easily obtained biological sources for biomarker discovery and screening, but all have associated limitations. Sputum is commonly used for the diagnosis of pulmonary tuberculosis, yet heavily immune-compromised adults and children can struggle to produce sputum. The major limitation to the use of plasma or serum for biomarker research is that non-specific responses from different effected tissues alter the composition of plasma during a disease state. A limitation of urine biomarkers for early diagnosis during sepsis-induced acute kidney injuries (AKI), is the low diuresis, making it difficult to study or use urine biomarkers in this case.

Thus, it appears that despite the limitations, plasma is the most relevant biological sample for the identification of early sepsis biomarkers (141). However, it is likely that the future of predicting the onset of AKI will entail the combination of multiple proteomics analyses of samples from urine, plasma (rather than serum), and tissue (142), because any alteration in the release of a given protein and the abundance of such a protein in a given environment could reflect a pathological state. For example, a study on the temporal profile of renal proteome changes induced by sepsis highlighted that ceruloplasmin (CR) and haptoglobin (Hp) are upregulated 90 min after the onset of sepsis. Proteins such as β-2-microglobulin (B2M) and α-1-antitrypsin (SERPINA1) increased in urine after sepsis-induced AKI (143), while levels of α-fibrinogen (FGA) chains are decreased. Another promising potential biomarkers are acidic nucleic protein deglycase DJ-1 (PARK7) and cadherin 16 (CDH16) (70). PARK7 functions as an inhibitor of cellular oxidative stress and a regulator of mitochondrial function, autophagy, and apoptosis (143), and CDH16 is exclusively expressed in the kidney (142).

The monitorization of concentration changes in proteoforms/proteins at high resolution also provides valuable personalized information, complementing traditional measurements of plasma concentrations of acute phase proteins (AAPs) for monitoring sepsis progression or, e.g., infectious endocarditis (IE) (144, 145). Proteoform profiles of individual glycoproteins, such as α-1-antichymotrypsin (AACT) are unique and therefore provides valuable personalized information complementing traditional measurements of plasma concentrations of APPs for monitoring sepsis progression (144). The host defense against infection is an individual adaptive response to decrease the pathogen load, limit tissue injury, and restore homeostasis (146). In addition, another set of techniques providing proof-of-concept evidence, are the new nanoscale-based “omics” enrichment technologies which substantially improve plasma proteomics analysis, uncovering novel biomarkers in a challenging clinical setting (136, 147). Proteins with stable and continuous changes are ideal biomarkers for the early identification and diagnosis of infectious disease (146).

As a final note, the identification and differentiation of antibiotic-resistant bacteria points toward the measurement of intact proteins by the means of MALDI-TOF, and especially to address to clinical diagnosis (1). However, until now, only a few biomarkers have been validated, because the prior LC separation adds a significant amount of time and complexity to the routine MALDI-MS analysis, which only reproducibly detects proteins > 20 kDa, thus excluding the possibility of detecting potential biomarkers known to exist below this mass range (148). Even so, different peaks have been associated to different mechanism of resistance, such as the peak *m/z* 4,594 presented as a doublet in MRSA isolates instead of a singlet, or the identification of the peak *m/z* 2,415 associated with phenol soluble modulin-mecA (PSM-mecA) (149), or the peak *m/z* 6,304 present in blaOXA-58 *Acinetobacter baumanii* isolates. The absence of the three peaks *m/z* 2,726; 5,455, and 5,742 is a characteristic of MDR *Pseudomonas aeruginosa* isolates or the non-peak *m/z* 6,100 in the carbapenem- and colistin-resistant isolates of *Klebsiella pneumonia* (150). Using LC-MALDI-ToF-MS, a protein biomarker at *m/z* 9,355 was identified which correlated with β-lactam resistance among the *Escherichia coli* (148). The analysis of proteotypic peptides by LC-ESI-MS/MS have identified porins (Opr) conferring multi-drug resistance and AmpC conferring resistance to cephalosporins and antipseudomonal penicillins (151).

## Concluding Remarks

The integration of proteomics with other “omic” technologies as well as computational approaches is a matter of fact. This integrative point of view provides research with opportunities to achieve a holistic picture of host–pathogen interactions, for a better understanding of the dynamics of infectious diseases and to discover future therapeutic targets. We admit that the integration of modern proteomics into pathogen studies (or even biomedical research in general) is far from where it should be at the time when this review is written. This is, at least in part, due to the fact that mass spectrometers still remain to be high-end instruments that require technical expertise, though ideally, they should appear commonly in the lab just as PCR machines. Finally, it is noteworthy that proteomics is a technology that is constantly evolving at a fast pace. We would certainly anticipate continued technical breakthroughs in the upcoming years, with which we will be able to gain much deeper insight into the biology of bacterial pathogens.

## Author Contributions

ET-S, AG, and CL performed the literature review, created figures and tables, and wrote the initial draft. ET-S, XL, and NS substantial contributed to the conception and design of the work. All authors contributed to drafting the work and revising it critically for important intellectual content, providing the approval for publication of the content, and the agreement to be accountable for all aspects of the work in ensuring that questions related to the accuracy or integrity of any part of the work are appropriately investigated and resolved.

## Conflict of Interest

The authors declare that the research was conducted in the absence of any commercial or financial relationships that could be construed as a potential conflict of interest.

## Publisher’s Note

All claims expressed in this article are solely those of the authors and do not necessarily represent those of their affiliated organizations, or those of the publisher, the editors and the reviewers. Any product that may be evaluated in this article, or claim that may be made by its manufacturer, is not guaranteed or endorsed by the publisher.
